# COVID-19: a vascular nightmare unfolding

**DOI:** 10.3389/fimmu.2025.1593885

**Published:** 2025-08-01

**Authors:** Qinan Yin, Youjin Huang, Hulin Wang, Yin Wang, Xuefei Huang, Yujie Song, Yueyuan Wang, Lizhu Han, Bian Yuan

**Affiliations:** ^1^ Department of Pharmacy, Personalized Drug Research and Therapy Key Laboratory of Sichuan Province, Sichuan Provincial People's Hospital, School of Medicine, University of Electronic Science and Technology of China, Chengdu, China; ^2^ Department of Vascular Surgery Center, Sichuan Academy of Medical Sciences & Sichuan Provincial People's Hospital, School of Medicine, University of Electronic Science and Technology of China, Chengdu, China

**Keywords:** COVID-19, thrombosis, mechanism, anticoagulation, vaccine

## Abstract

The emergence of COVID-19 has been associated with an increased risk of arteriovenous thrombosis, with immune inflammation playing a significant role in the pathogenesis of thrombosis. Numerous drug-related clinical trials have been undertaken to prevent thrombosis, and guidelines for its prevention and treatment are continuously evolving as our understanding of the disease progresses. This article provides a comprehensive review of the mechanisms underlying thrombosis in COVID-19 patients, as well as the advancements in clinical trials and guidelines for thrombosis prevention with pharmacological interventions.

## 1 Introduction

**Figure 1:**
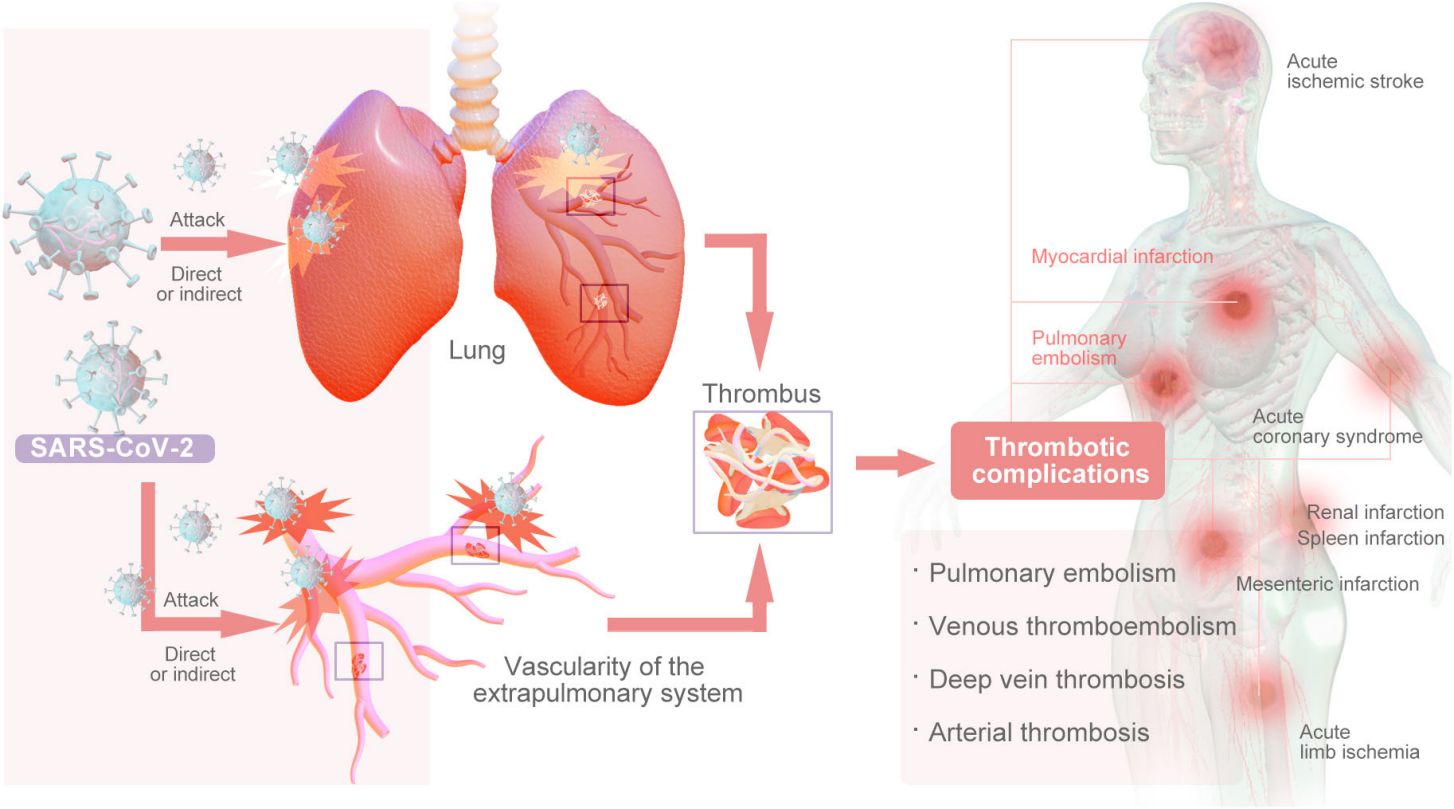
The Main Types of Thrombsiss Caused by COVID-19.

COVID-19, caused by the coronavirus SARS-CoV-2, is primarily a respiratory viral infection; it has had a catastrophic impact on the world’s population, resulting in more than 6.5 million deaths worldwide. This represents the worst global health crisis since the 1918 influenza pandemic (1). The primary impact of SARS-CoV-2 infection is on the pulmonary system, however, emerging evidence suggests that it may also have implications for the vascularity of the extrapulmonary system, either through direct viral infection or indirectly through cytokine storms (2). Studies have shown that COVID-19 induces a prethrombotic state (3). Thrombosis of both microvessels and large vessels is prevalent among COVID-19 patients (4). Pulmonary embolism (PE) and deep vein thrombosis (DVT) are frequently observed thrombotic complications in patients with COVID-19. Arterial thrombosis is a notable occurrence in COVID-19 patients, leading to various complications, such as acute ischemic stroke, acute coronary syndrome (ACS), acute limb ischemia (ALI), mesenteric infarction, renal infarction, and spleen infarction (**Figure 1**) (5, 6).

High mortality is associated with hypercoagulability in COVID-19 patients (7). A recent study revealed that patients with COVID-19 are more likely to develop venous thromboembolism (PE: 1.0–40.0%, DVT: 0.4–84%) than arterial thromboembolism (stroke: 0.5–15.2%, myocardial infarction: 0.8–8.7%). Finally, the all-cause mortality rate for COVID-19 patients ranges from 4.8% to 63%, whereas the mortality rate associated with thromboembolic complications ranges from 5% to 48% (8). A systematic review and meta-analysis of thromboembolic events in patients with COVID-19 from the start of the pandemic through August 31, 2021, involving 63 studies (104,920 patients with COVID-19) revealed an overall thrombotic rate of 21% (95% CI, 18%–25%). The deep vein thrombosis rate was 20% (95% CI, 16% ~ 25%), the pulmonary embolism rate was 8% (95% CI, 6% ~ 10%), and the arterial thrombosis rate was 5% (95% CI, 3% ~ 7%). The prevalence of all major outcomes in critically ill patients in the intensive care unit (ICU) significantly increased (P<0.05). The incidence of total thrombosis, pulmonary embolism and deep vein thrombosis in elderly patients significantly increased (P<0.05) (9). Furthermore, despite the implementation of anticoagulation prophylaxis, hospitalized individuals continue to face increased susceptibility to venous thromboembolism (VTE) (4). Almost all of the above findings are based on hospitalized COVID-19 patients. These findings are based on populations in an inpatient setting, but data on the incidence of venous thromboembolism events, such as acute pulmonary embolism (PE) and deep vein thrombosis (DVT), among individuals recovering from COVID-19 are limited. A systematic review and meta-analysis were conducted to assess the risk of acute PE and DVT in this population. The study included a total of 29,078,950 patients, with a mean age of 50.2 years, 63.9% of whom were male. Among these, 2,060,960 patients had been infected with COVID-19. The cumulative incidence rates of PE and DVT in patients who had recovered from COVID-19 were 1.2% (95% CI: 0.9–1.4, I: 99.8%) and 2.3% (95% CI: 1.7–3.0, I: 99.7%), respectively. During the same follow-up period, the hazard ratios for developing PE and DVT in recovered COVID-19 patients were 3.16 (95% CI: 2.63–3.79, I: 90.1%) and 2.55 (95% CI: 2.09–3.11, I: 92.6%), respectively (10). A separate study incorporated inpatient participants with primary care records from the Netherlands, Italy, Spain, and the United Kingdom, as well as outpatient specialist records from Germany. The 90-day cumulative incidence of venous thromboembolism (VTE) in COVID-19 patients ranged from 0.2% to 0.8%, increasing to as high as 4.5% among hospitalized patients. The incidence of VTE in COVID-19 patients was correlated with an elevated risk of mortality, with an adjusted hazard ratio (HR) of 4.42 [95% CI: 3.07–6.36] for nonhospitalized patients and 1.63 [95% CI: 1.39–1.90] for hospitalized patients. Similarly, the development of arterial thromboembolism was linked to an increased risk of death, with hazard ratios of 3.16 [95% CI: 2.65–3.75] for nonhospitalized patients and 1.93 [95% CI: 1.57-2.37] for hospitalized patients (11). Overall, the prevalence of COVID-19 was greater in women than in men. In a 2022 European network cohort study involving 909,473 COVID-19 patients and 32,329 COVID-19 hospitalizations, there were more COVID-19 cases in women than in men across all databases (11). A 2024 systematic review and meta-analysis also reported that the prevalence of post-COVID-19 syndrome by sex was 47.23% (95% CI: 44.03–50.42%) in men and 52.77% (95% CI: 49.58–55.97%) in women (12). For patients with COVID-19 and thrombosis, a prospective study identified 1106 patients with venous thromboembolism associated with COVID-19 (age, 62.3 ± 14.4 years; 62.9% male) (12, 13). Interestingly, women appear to be less likely to develop severe disease, despite the data collected thus far that more women are affected and that the mortality rate in women is lower than that in men. The role of female hormones in regulating inflammation may be the reason behind this sex difference.

At present, the association between COVID-19 and thrombosis and related anticoagulation therapy has received extensive attention. This article summarizes and explores the pathogenic mechanism of thrombosis induced by COVID-19 and evaluates current anticoagulation strategies for the prevention and management of COVID-19-associated thrombosis, providing a reference for the clinical formulation of scientific anticoagulation strategies.

## 2 Mechanism of thrombosis

**Figure 2:**
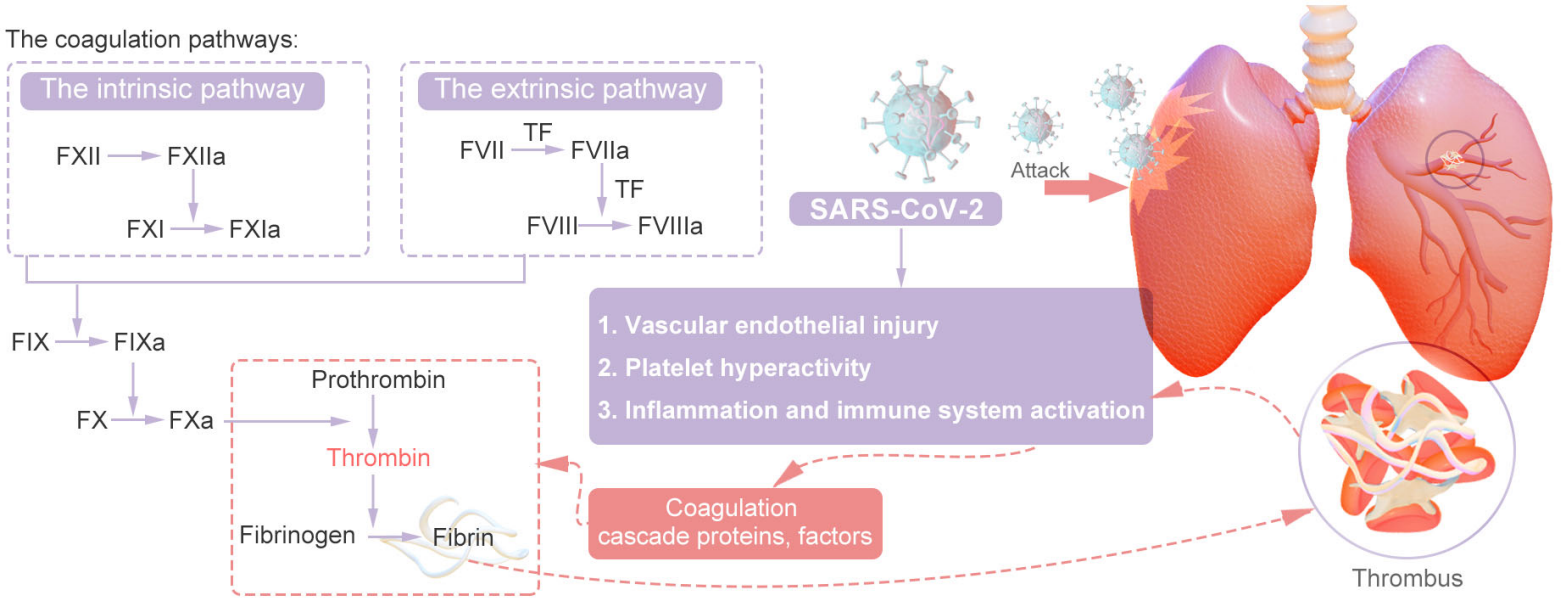
Simple Mechanism of COVID-19 Thrombosis.

**Figure 3:**
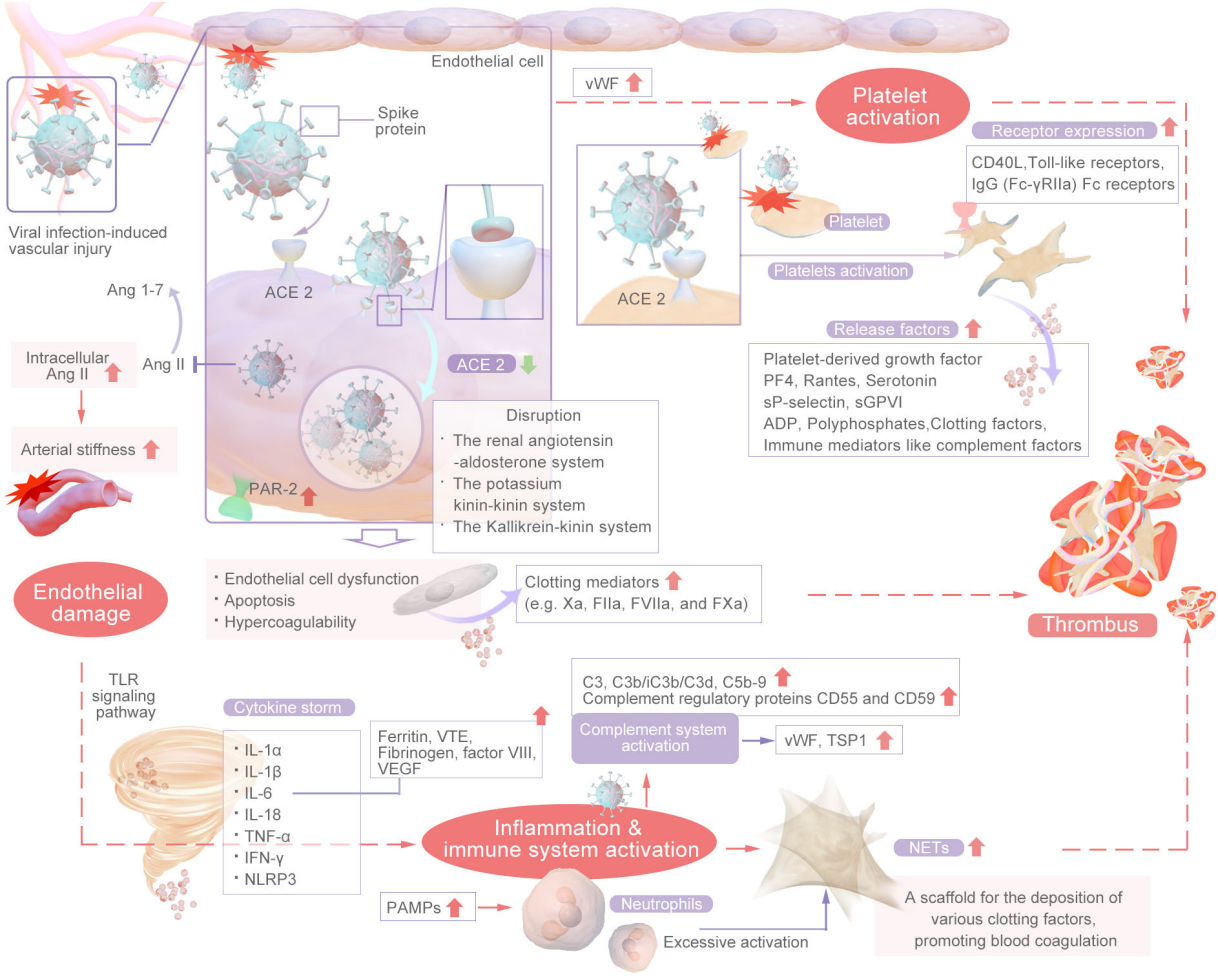
The Main Mechanism of COVID-related Thrombosis.

Thrombosis is usually associated with vascular injury, blood stasis, and changes in blood properties. Coagulation involves a series of enzymatic reactions leading to thrombin production and fibrin formation (14). The coagulation pathways include two main pathways: the extrinsic pathway and the intrinsic pathway. In addition, the common pathway converges on factor X (FX). The coagulation pathway activates coagulation factors through a cascade of activation, also known as the coagulation cascade. The extrinsic pathway is the main structural element of the coagulation process; components of the extrinsic and common pathways are essential for coagulation, whereas components of the intrinsic pathway are needed to amplify thrombin production. Repeated activation and amplification trigger the intrinsic pathway driven by thrombin (under certain conditions driven by FXIIa) to accelerate thrombin and fibrin formation. Both the extrinsic and intrinsic pathways of the coagulation system are initially triggered by the release of active tissue factor (TF), the formation of TF complexes with FVIIa, and the subsequent activation of FX directly to FXa or through the FIXa pathway (15). Certain scholars posit that SARS-CoV-2 could exacerbate thrombosis by exerting various distinct and comprehensive influences on intravascular dysfunction, platelet activation and aggregation, the initiation of the coagulation cascade, the activation of eicosanoid pathways, and the disruption of fibrinolysis (16–20). Research has revealed that the levels of circulating inflammatory markers (CCL23 and IL-6) and vascular dysfunction markers (ACE-2 and TF) are significantly greater in individuals with severe illness than in those with mild illness (21). Recent research suggests that the mechanisms underlying thrombosis in individuals with COVID-19 are primarily initiated by vascular endothelial damage, dysregulation of the body’s coagulation system, the presence of viral particles, and subsequent immune responses. This process is multifactorial in nature (22, 23). In summary, inflammation facilitates thrombosis, which subsequently exacerbates the inflammatory response. Inflammation alters the functional phenotype of endothelial cells from an antithrombotic to a thrombotic state, thereby promoting the activation of the coagulation cascade. Thrombin generated during thrombotic events further amplifies inflammation by stimulating cells that express protease-activated receptors (PARs) to secrete cytokines. Furthermore, activated platelets engage with inflammatory and endothelial cells, enhancing neutrophil extracellular trap (NET) formation and cytokine release (24). The main mechanisms are shown in **Figures 2**, **3**.

### 2.1 Vascular endothelial injury

SARS-CoV-2 affects endothelial cells through multiple mechanisms. Autopsies of patients with SARS-CoV-2 infection have shown evidence of endothelial cell apoptosis. The microvascular endothelial damage and microcirculation thrombus formation found in the autopsy assessment are consistent with the possible thrombotic microangiopathy that the patients may have (16). The study also found that circulating endothelial cells (CEC), a cellular biomarker derived from damaged blood vessels, were elevated in patients with severe COVID-19 and in those with underlying conditions. Notably, even patients who recovered from COVID-19 had significantly higher numbers of circulating endothelial cells than healthy individuals, suggesting that vascular dysfunction persists after recovery from COVID-19 (25).

**Figure 4:**
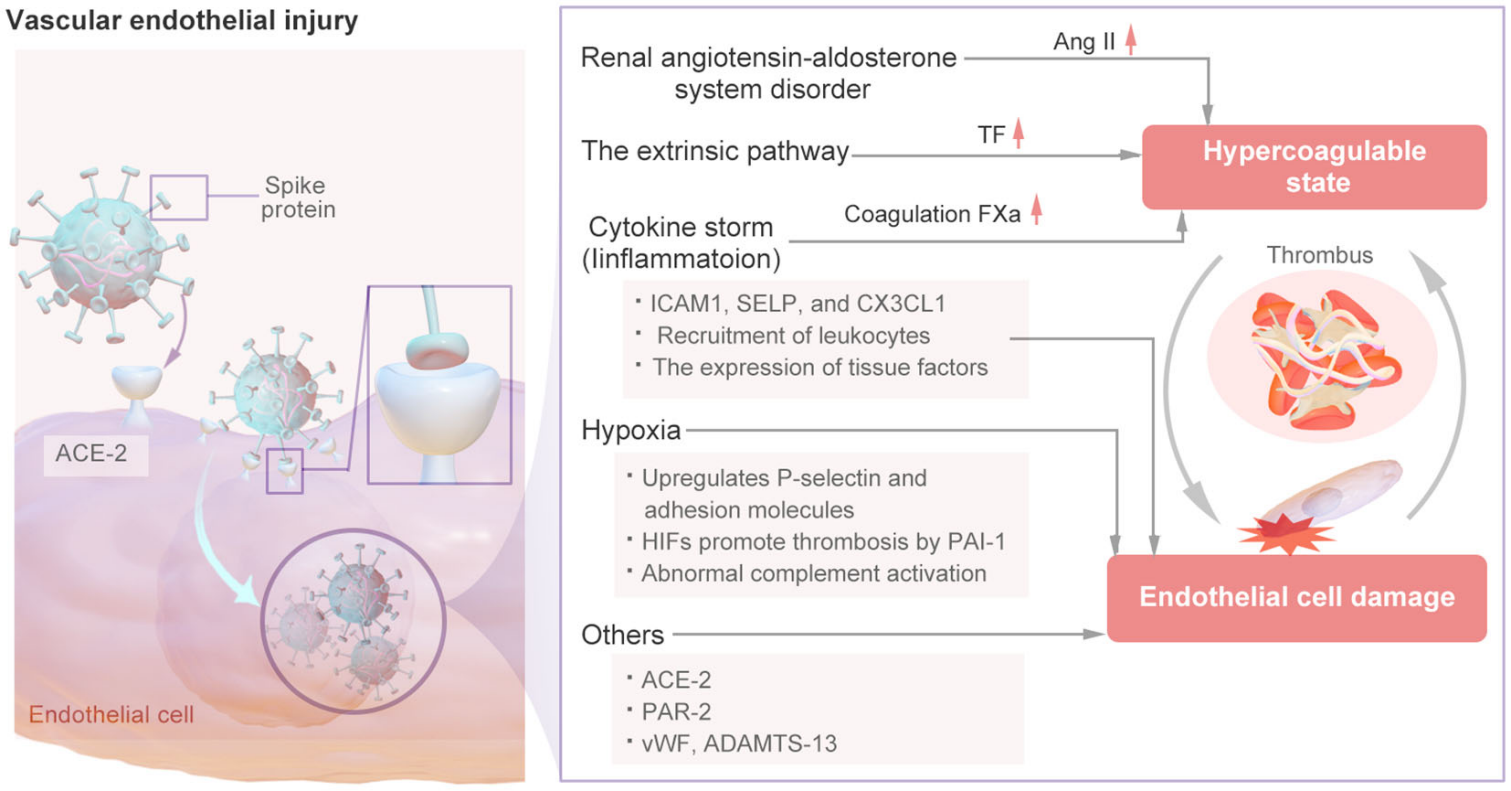
The Mechanism of Vascular Endothelial Injury.

The mechanism of vascular endothelial injury is shown in **Figure 4**. In the renin–angiotensin system (RAS), Ang I is converted into Ang II by ACE-2 (26). Ang II-mediated AT1R activation can increase platelet activation and prevent fibrinolysis, resulting in a hypercoagulable state (27). SARS-CoV-2 has been confirmed to have a direct effect on alveolar and systemic endothelial cells, causing endothelial dysfunction, altering the microvascular balance, and subsequently leading to organ ischemia, a procoagulation state, tissue inflammation and edema (28). SARS-CoV-2 exhibits a distinct affinity for the angiotensin-converting enzyme 2 (ACE2), which serves as its cellular receptor. This receptor is expressed by type II alveolar cells and is situated in close proximity to the pulmonary vascular network (29). The spike (S) proteins of SARS-CoV-2 bind to angiotensin-converting enzyme-2 (ACE-2) on endothelial cells, leading to endothelial cell damage (3). When SARS-CoV-2 binds to cells by ACE-2, the pulmonary expression of membrane-bound ACE-2 may be downregulated, leading to the accumulation of Ang II within cells. Ang II has various proinflammatory and prothrombotic effects and can be amplified in patients with COVID-19 (4). Thus, SARS-CoV-2 directly infects vascular endothelial cells, leading to cell damage and apoptosis, thereby reducing the antithrombotic activity of normal endothelial cells.

Viral infection-induced vascular injury may stimulate endothelial cells to increase the expression of TF and activate the extrinsic coagulation pathway (30), Additional cytokines may contribute to the induction of tissue factor (TF) expression in endothelial cells derived from COVID-19 patients, thereby initiating the extrinsic coagulation pathway (31). The interaction between the viral spike protein and the cellular receptor can trigger the inflammatory pathway, alter the mitochondrial metabolism of microvascular endothelial cells, and induce significant production of coagulation factor Xa, resulting in a hypercoagulable state (32, 33). this also leads to excessive thrombin production and reduced fibrinolysis, and thrombin can cause further endothelial damage (34, 35). Moreover, The increased expression of ICAM1, SELP, and CX3CL1 may suggest that endothelial cells from the site of vascular injury are in a proinflammatory and procoagulant state (36). serum from patients with COVID-19 can modify the functional characteristics of endothelial cells through the activation of protease-activated receptor 2 (PAR-2), leading to increased apoptosis, compromised barrier integrity, and increased propensity for blood clotting (37). Moreover, endothelial dysfunction is facilitated by PAR-2, whereby certain clotting mediators (e.g., FIIa, FVIIa, and FXa) can trigger the production of proinflammatory cytokines, thus perpetuating a detrimental cycle (38). Endodermatitis induced by SARS-CoV-2 results in the release of elevated levels of vWF, a glycoprotein originating from activated endothelial cells, platelets, or subendothelial cells that plays a crucial role in mediating platelet adhesion and aggregation. The significant increase in vWF levels observed in individuals with COVID-19 signifies endothelial damage or impairment, promoting platelet aggregation at the site of injury and potentially leading to hyperactivation of the coagulation pathway, thereby initiating thrombotic events (39–41). Concurrently, a reduction in a disintegrin and metalloproteinase with a thrombospondin type 1 motif, member 13 (ADAMTS-13) levels was noted, suggesting an imbalanced vWF: ADAMTS-13 ratio that could contribute to thrombotic occurrences. Both the vWF/ADAMTS-13 ratio and PF4 level are increased, indicating that prolonged endothelial cell activation leads to increased platelet aggregation after COVID-19 (42, 43).

COVID-19 patients often suffer from severe hypoxia, and hypoxia can also lead to endothelial dysfunction and coagulation. Hypoxia upregulates P-selectin and adhesion molecules (such as intracellular adhesion molecule-1) to induce the recruitment of platelets and leukocytes (44). Furthermore, hypoxia-inducible transcription factors (HIFs) promote thrombosis by increasing the release of the inflammatory cytokine PAI-1 by endothelial cells while downregulating thrombomodulin expression (45). Specifically, HIF-α downregulates the expression of the complement regulator CD55. This downregulation increases the release of C3a and the deposition of caspase 3 on endothelial cells, thereby further increasing complement-mediated endothelial damage in patients with COVID-19. Damaged endothelial cells subsequently trigger hypercoagulability (46). The abnormal activation of endothelial cells triggered by SARS-CoV-2 can further lead to cytokine storm and activation of the complement system, as described in the following sections.

### 2.2 Inflammation and immune system activation

**Figure 5:**
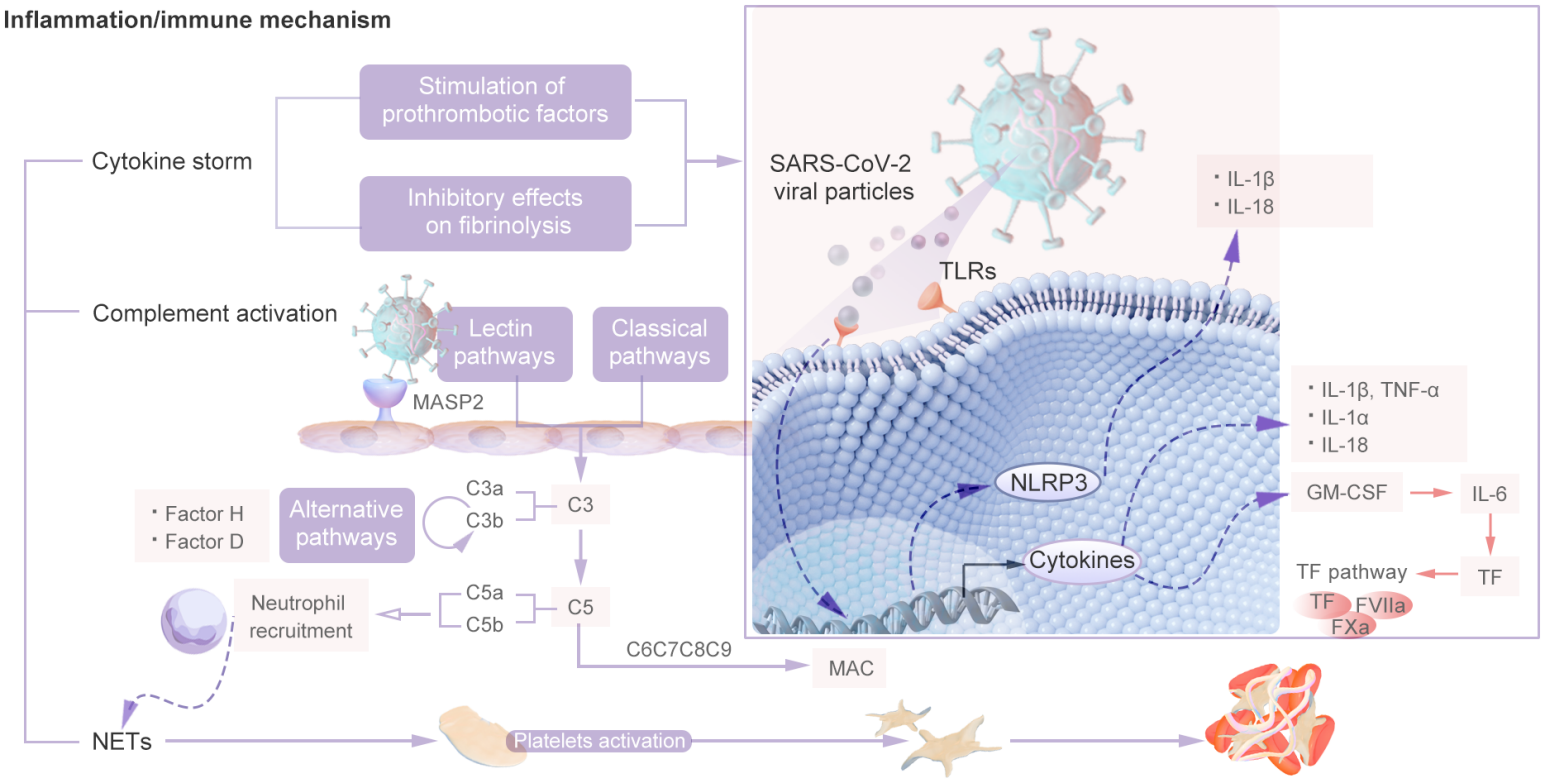
The Inflammatory and Immune Thrombosis Related Mechanisms.

COVID-19-associated coagulopathy may be a downstream consequence of the host inflammatory response to SARS-CoV-2 and of innate immune activation (4). During the course of COVID-19, the recruitment of immune cells and the subsequent production of proinflammatory cytokines serve as mediators of clotting activation in tissues affected by the virus. Viral infection triggers an inflammatory response in the host, leading to an increase in the production of proinflammatory cytokines. These cytokines have pleiotropic effects, including activation of coagulation and thrombin generation, and this phenomenon is known as thromboinflammation or immunothrombosis. The main mechanisms are cytokine storm, complement system activation and neutrophil extracellular traps (NETs), which constitute the “malignant triangle” of immune-related thrombosis. The inflammatory and immune thrombosis-related mechanisms is shown in **Figure 5**.

#### 2.2.1 Cytokine storm

Cytokine storm syndrome has been documented in 10-20% of patients with COVID-19 pneumonia and may result in severe outcomes, including multiorgan failure and mortality (47). The cytokine storm and the consequent hyperinflammatory state determine local and systemic consequences, inducing arterial and venous vasculopathy in the lung with thrombosis of the small vessels and progression toward serious lung lesions, acute respiratory distress syndrome (ARDS) and, in some cases, disseminated intravascular coagulation (DIC) (48, 49). SARS-CoV-2 infection in the host lungs, upon entry through type 2 alveolar epithelial cells and interaction with blood vessels at the pneumocyte–capillary interface, causes the activation of host innate immunity, leading to the release of proinflammatory cytokines, which eventually results in an uncontrolled and exaggerated response, the cytokine storm (50). Cytokine storm syndrome is associated with major thrombotic events in patients with COVID-19, and the mechanism is related primarily to the simultaneous stimulation of prothrombotic factors and inhibitory effects on fibrinolysis (28).

The activation of Toll-like receptors (TLRs) by molecules present in SARS-CoV-2 viral particles initiates the innate immune response. However, excessive activation of TLRs can disrupt intracellular signaling pathways, exacerbate the inflammatory response, and lead to an excessive and uncontrolled release of cytokines (51). Firstly, activated macrophages and monocytes release a large amount of pro-inflammatory cytokines such as IL-6, (IL)-1β, and TNF-α (52). In addition, Granulocyte-macrophage colony-stimulating factor (GM-CSF) is a cytokine that facilitates the activation and mobilization of myeloid cells to inflammatory sites. It is synthesized by a variety of cell types. In patients with COVID-19, the production of GM-CSF initiates a positive feedback loop, leading to the overexpression of interleukin-6 (IL-6) and other proinflammatory factors, particularly by CD14+ and CD16+ inflammatory monocytes (53). In particular, TNF-α and IL-6 levels increase to levels that are not typically observed in patients with bacterial sepsis or influenza (54). In the context of inflammation, macrophages are stimulated to release TF in response to IL-6. The upregulation of the expression of IL-6 and its receptor in COVID-19 can lead to increased activation of endothelial cells, resulting in the excessive release of TF. This cascade of events can contribute to infection-induced coagulation dysfunction and increased platelet proliferation (55, 56). IL-6 facilitates the secretion of various cytokines, activates immune cells, and contributes to the synthesis of specific coagulation factors, including fibrinogen and factor VIII. (57). Moreover, IL-1α is involved in the activation of the inflammatory cascade in thrombotic pathology and serves as a crucial factor in thrombosis through the recruitment of granulocytes, prolonging clot lysis time and increasing platelet activity (50). Furthermore, the NOD-like receptor thermal protein domain associated protein 3 (NLRP3) inflammasome is responsible for the generation of six primary proinflammatory cytokines belonging to the interleukin-1 family, namely, IL-1β and IL-18. NLRP3 inflammasome activation also increases immune thrombosis through mechanisms such as the release of neutrophil extracellular traps and TF by leukocytes, as well as the initiation of prothrombotic responses in platelets and the vascular endothelium (58). Therefore, it has been suggested that cytokine release may also be the result of high levels of extracellular neutrophil traps.

#### 2.2.2 Complement activation

The complement system serves as the primary defense mechanism against pathogen invasion and damage to host cells and consists of a complex network that includes over 40 soluble and membrane-binding proteins. This finely tuned response is typically maintained at low levels of activity in individuals who are in good health, allowing for a rapid and localized response to insults as they arise. The complement system operates through three distinct pathways—classical, lectin, and alternative—to provide comprehensive protection against invading pathogens (59). The complement system serves as the primary immune response of the host to infection, but uncontrolled activation can lead to increased cellular damage, inflammation, and intravascular clotting and ultimately results in multiple organ failure and mortality (60).

patients with SARS-CoV-2 infection, complement system dysregulation has been observed, which is primarily characterized by disturbances in the terminal complement system and the persistent activation of both the alternative and classical complement pathways (39). The NP of SARS-CoV-2 can bind to mannose-binding lectin serine protease 2 (MASP 2), a key component of the complement system, which exacerbates tissue damage and leads to the release of inflammatory factors, thereby increasing cell adhesion and thrombosis during long-term COVID-19 (61). In instances of thrombotic inflammation, the complement pathway may initiate the coagulation cascade through the induction of tissue factor expression. The activation of the complement cascade may also recruit and activate white blood cells, leading to the local release of the proinflammatory cytokines IL-1, IL-6, IL-8 and interferon gamma, which are significantly amplified and subsequently cause microvascular damage (4). Compared with normal lung tissues, the lung tissues of COVID-19 patients display complement system hyperactivation, characterized by extensive deposition of C3, C3b/iC3b/C3d, and C5b-9 and the increased expression of the complement regulatory proteins CD55 and CD59 (62). In Addition, serine proteases within the lectin pathway are capable of cleaving prothrombin to generate activated thrombin. This mechanism is particularly evident in critically ill COVID-19 patients experiencing symptomatic thromboembolism, where there is a marked elevation of mannose-binding lectin (63). Research has demonstrated that the SARS-CoV-2 spike protein activates the complement alternative pathway by disrupting the function of complement factor H, a critical negative regulatory component of this pathway. Inhibitors targeting C5 and factor D have been shown to effectively prevent the accumulation of C5b-9 complexes induced by the SARS-CoV-2 spike protein (64). The activation of the complement stem in COVID-19 patients results in the formation of a terminal complement complex (TCC) consisting of C5b-9. Among them, C5b-9 is regarded as one of the key factors affecting the hemostasis system in the quantitative system immunology model (65). Furthermore, the insertion of the complement C5b–C7 complex into the endothelial cell membrane results in cellular damage, which subsequently triggers the release of vWF and thrombospondin-1 (TSP1). This process is highly prothrombotic, as the resulting vWF polymers facilitate platelet recruitment and thrombin generation while simultaneously inducing C3b binding and activating alternative complement pathways. This feedback mechanism perpetuates localized complement activation and inflammation and the formation of microthrombi (66). C5a plays a pivotal role in the recruitment and activation of neutrophils, monocytes, and macrophages, while simultaneously enhancing the expression of adhesion molecules on endothelial cells and platelets. This process can result in elevated production of interleukin-6 and CXCL8, subsequently initiating a robust procoagulant response. Consequently, this leads to the formation of neutrophil-derived tissue factors and the development of the neutrophil extracellular trap (67). Furthermore, the complement components C5a and the membrane attack complex facilitate platelet adhesion by inducing the secretion of endothelial P-selectin and vWF polymers, increasing the expression of TF and promoting platelet shedding, thereby contributing to thrombogenesis (68, 69).

#### 2.2.3 Neutrophil extracellular traps

Neutrophils, also known as polymorphonuclear (PMN) cells, constitute the first line of defense in the innate immune system against invading pathogens. PMNs are the most abundant white blood cells in the human body and perform multiple protective functions, including phagocytosis of pathogens, degranulation, secretion of cytokines, and formation of NETs (70). Neutrophil extracellular traps (NETs) are complex, web-like structures consisting of DNA, histones, and antimicrobial proteins, which are extruded by activated neutrophils to ensnare and neutralize pathogens. Neutrophil elastase (NE), a constituent of neutrophil extracellular traps (NETs), plays a crucial role in platelet activation via the protease-activated receptor 4 (PAR4) and thrombin pathways, consequently altering platelet functionality and promoting fibrinogenesis. Additionally, myeloperoxidase (MPO), another integral component of NETs predominantly secreted by neutrophils, serves as a primary scavenger of the pro-oxidant hydrogen peroxide (71). The procoagulant properties of NETs are primarily attributed to their filamentous structures, which increase the adhesion of activated platelets and subsequently facilitate the entrapment of red blood cells (72).

Excessive NET formation is a process known as NETosis, which can occur in patients with COVID-19 and other viral infections, and can lead to hypercoagulation and thrombosis (73). Research findings spleen tyrosine kinase (Syk), in conjunction with C-type lectin member 2 (CLEC2), which is predominantly expressed in platelets and alveolar macrophages, is capable of directly interacting with the receptor binding domain (RBD) of the SARS-CoV-2 spike protein. In contrast to filamentous neutrophil extracellular traps (NETs), SARS-CoV-2 facilitates the formation of aggregated NETs in the presence of wild-type (WT) platelets; however, this aggregation is absent in CLEC2-deficient platelets. Subsequent investigations have demonstrated that a SARS-CoV-2 spike protein pseudolentivirus can induce NET formation via CLEC2, suggesting that the SARS-CoV-2 RBD promotes NET formation by binding to CLEC2, thereby activating platelets (74). High levels of extracellular neutrophil traps can also contribute to the release of cytokines, which play crucial roles in thrombotic events in critically ill patients (73, 75). Significantly, NETs exacerbate lung damage by triggering epithelial cell and endothelial cell death, leading to the secretion of cytokines such as IL-1β and IL-6, which initiate cytokine storms that exacerbate thrombosis (76). An intensified cycle of systemic and neutrophil-derived interleukin-8 (CXCL8/IL-8) dysregulation initiates and sustains neutrophil-driven immunopathology. This systemic positive feedback loop of neutrophil autocrine IL-8 results in an activated pre-thrombotic neutrophil phenotype, marked by degranulation and the formation of neutrophil extracellular traps (NETs). In severe cases of COVID-19, neutrophils directly trigger coagulation and complement cascade reactions, underscoring their association with the immune thrombotic status observed in these patients (77). A study examining NETs and platelet involvement in the autopsies of lungs from 3 COVID-19 patients revealed that guanidino histone H3+ neutrophils might undergo NETosis and colocalize with platelet-derived thrombogenic factors in pulmonary microthrombi (78). The findings indicate that the interaction between neutrophil extracellular traps (NETs) and platelets in patients with COVID-19 may initiate a thrombogenic cascade, culminating in a hypercoagulable state and subsequent thrombosis. Research has demonstrated that NETs play a role in promoting the thrombotic factor vWF in a platelet-dependent fashion and directly activate the clotting cascade through mechanisms including platelet adhesion activation, cell–fibrinogen binding, and von Willeophilia. Furthermore, NETs have been found to contribute to local thrombin production, thereby increasing the risk of thrombosis in addition to their effect on primary hemostasis (79). Furthermore, the colocalization of NETs with factor XII was confirmed, suggesting that NETs may serve as a platform for the activation of the intrinsic coagulation pathway involving factor XII (80).

### 2.3 Platelets

#### 2.3.1 Platelet activation

**Figure 6:**
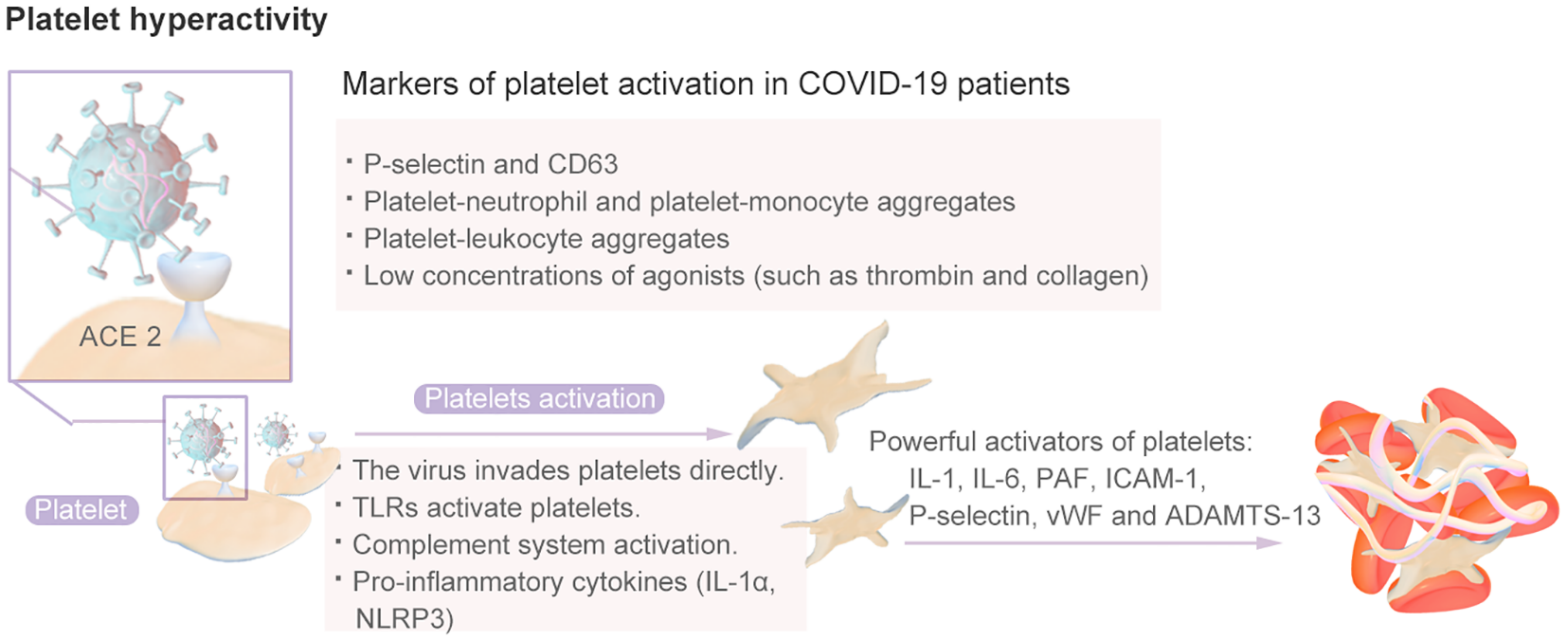
The Platelet Hyperactivity Mechanisms.

Platelets have long been recognized for their crucial role in thrombi and the hemostatic system, as they rapidly respond to pathogens, signaling nearby immune cells and facilitating thrombosis and intravascular clot formation (81). The inflammation caused by viral infection can stimulate platelets to express TF, thereby promoting the interaction between platelets and monocytes and the formation of more platelet–monocyte aggregates that promote coagulation (82). Platelets from COVID-19 patients exhibit a hyperactive phenotype (83). Compared with those from healthy individuals, platelets from COVID-19 patients, including those with severe disease, are more sensitive to aggregation in response to low concentrations of agonists, such as thrombin and collagen (17). A high proportion, approximately 90%, of individuals with COVID-19 were shown to exhibit increased platelet aggregation, elevated platelet activation, platelet–monocyte aggregation and formation, and increased levels of fibrinogen and collagen diffusion (84). Therefore, SARS-CoV-2 infection induces greater platelet reactivity. The platelet hyperactivity mechanisms is shown in **Figure 6**. Research has indicated that hyperplatelet activity persists for a minimum of 40 days in the majority of COVID-19 patients, irrespective of the severity of the disease, following the resolution of acute inflammation. The sustained elevation in platelet activity after recovery from COVID-19 may predispose patients to thromboembolic complications (85).and results in an increased platelet reaction in the context of this particular illness, a phenomenon known as “fibrinolysis shutdown” (82).

Various mechanisms explain platelet activation in patients with COVID-19. The first mechanism is related to the inflammatory response to SARS-CoV-2 infection and the resulting cytokine storm and endothelial dysfunction (82). IL-1, IL-6, platelet-activating factor (PAF), intercellular adhesion molecule 1 (ICAM-1), P-selectin and vWF released by endothelial cells are all powerful activators of platelets that increase the binding of platelets to endothelial cells, thereby further inducing platelet activation and aggregation and leading to a progressively stronger effect; this mechanism may even induce DIC and thrombocytopenia. SARS-CoV-2 can enter endothelial cells through the ACE-2 receptor, CD147, toll-like receptors (TLRs), or C-type lectin-like receptor 2 (CLEC-2) and platelets through the pericellular region. Subsequently, platelet activation leads to the release of platelet granules, the overexpression of TF, the formation of platelet leukocyte aggregates, and the formation of NETs (86).Moreover, the imbalance between vWF and ADAMTS-13 is related to platelet activation. An elevated vWF/ADAMTS-13 ratio may indicate an acquired ADAMTS-13 deficiency, which results in the retention of larger circulating vWF molecules and increases the risk of platelet binding and microthrombosis (82). Once activated, platelets secrete a vast repertoire of bioactive molecules, such as adenosine diphosphate (ADP), thromboxane A2 (TXA-2) and proinflammatory cytokines, from their intracellular granules that play essential roles in thrombus formation and inflammation (87). There is evidence that increased platelet response during SARS-CoV-2 infection is, at least in part, mediated by increased mitogen-activated protein kinase (MAPK) signaling. The activation of MAPK in platelets induces the activation of cytosolic phospholipase A2 (cPLA2), which results in thromboxane synthesis (88).

Sera from patients with severe COVID-19 possess the capability to activate platelets through the cross-linking of their FcγRIIa receptors. Such sera can induce a procoagulant phenotype in platelets derived from healthy donors. This mechanism can be triggered not only by antibody-mediated pathways but also by immune complexes (89). Immune complexes, which are soluble antigens formed through the binding of antibodies, serve as a crucial initial defense mechanism against pathogenic infections. In the context of COVID-19, these complexes consist of spike proteins from the virus and their corresponding antibodies. The interaction between immune complexes and platelet receptors triggers the activation of platelets and the release of intracellular molecules, including serotonin. This particular immune complex facilitates platelet activation and thrombosis in COVID-19, resembling the pathophysiological process observed in heparin-induced thrombocytopenia (90). Research has elucidated the mechanism by which the SARS-CoV-2 S1 spike protein (S1) induces platelet activation. Specifically, IgG antibodies bind to the S1 protein to form immune complexes, which enhance the expression of the FcγRIIa receptor on platelets, facilitating receptor cross-linking. This process initiates platelet activation and aggregation, leading to the formation of platelet-leukocyte aggregates (PLAs). Consequently, this mechanism directly contributes to the prothrombotic environment observed in COVID-19 (91).

#### 2.3.2 Procoagulant platelets

Procoagulant platelets are a subset of activated platelets that expose phosphatidylserine (PS) and promote thrombin production (92). Studies have shown that the ΔΨm depolarization, cytosolic Ca2+ and PS externalization levels of platelets from COVID-19 patients in the ICU are high and that the expression of apoptosis markers is upregulated. Serum and immunoglobulin (IgG) fractions isolated from COVID-19 patients can induce the apoptosis of platelets from healthy donors, followed by changes in the coagulation system (93). Similarly, another study demonstrated a significant elevation in the levels of phosphorylated AKT in the platelets of COVID-19 patients, which was associated with CD62p expression and PS externalization. Notably, procoagulant thrombopoiesis induced by serum from COVID-19 patients in the ICU occurs independently of GPllb/llla. Furthermore, the inhibition of AKT and PI3K protein phosphorylation effectively prevented the production of procoagulant platelets. Thus, targeting PI3K/AKT phosphorylation may represent a promising therapeutic strategy to mitigate the risk of thrombosis in patients with severe COVID-19 (94).

### 2.4 Extracellular vesicle mechanism

Extracellular vesicles (EVs) are lipid bilayer-enclosed particles secreted by cells, capable of transferring proteins, lipids, metabolites, nucleic acids, and organelles to other cells, and are typically found in distant tissues throughout the body. EVs exhibit considerable heterogeneity, with sizes ranging from 20 nanometers (nm) to 1 micrometer. They can be categorized based on size, with exosomes representing the smaller end of the spectrum and microvesicles (formerly referred to as microparticles) representing the larger end. EVs are released by various cell types, including platelets, megakaryocytes, white blood cells, endothelial cells, and red blood cells, and these cells may be influenced by the effects mediated by EVs. EVs perform multiple functions, such as facilitating cell-cell communication, removing cellular waste or recycling molecules, and mediating host-pathogen interactions. Platelet-derived EVs may originate from the plasma membrane of resting platelets or from α granules following platelet activation. EVs derived from endothelial cells and monocytes express selectin, TF, vWF, other coagulation factors, and negatively charged phosphatidylserine, all of which can contribute to thrombosis. Additionally, circulating EVs may play a role in regulating inflammation and vascular permeability (95, 96).

Current studies have confirmed that extracellular vesicles (EVs) promote integrates the triple functions of “coagulation - inflammation - viral transmission”. Different from simple NETs or cytokine storm in COVID-19 (97). Studies have shown that the activity levels of EV-related TF in COVID-19 patients are higher than those in the control group (98). The activity of EV-related TF is closely related to the D-dimer level, which is a marker of thrombosis in this patient group. The EV level is also associated with prothrombin time, fibrinogen level, plasmin-antiplasmin complex, von Willebrand factor and ADAMTS13 (98). Another study compared severely ill COVID patients with non-COVID patients with septic shock and found that COVID-19 patients had a higher pro-coagulant effect, and the EV-TF activity was significantly higher (99).

EVs facilitate the coagulation process via the exposure of phosphatidylserine, providing a catalytic surface to facilitate the formation of the tenase (factors VIIIa, IXa, and X) and prothrombinase (factors Va, Xa, and II) complexes of the coagulation cascade (100). And EVs initiate clotting by surface expression of tissue factor (TF) under inflammatory conditions (101). Studies have characterized the expression of TF in clinical samples from patients with severe COVID-19, and it was found that the ratio of TF to EV was greater than 15% (101).

Moreover, Endothelial cells (EC) can release EVs (EC-EVs) that express endothelial markers CD31 (102). ECEC-EVs play a relevant role in regulating the survival of ECs, and they also participate in triggering coagulation and complement cascade reactions (103).

## 3 Prevention and treatment of thrombosis

Coagulation dysfunction and microvascular and macrovascular thrombotic events are common features of patients with acute COVID-19 in the early stages of the pandemic. Over the past four years, the incidence and manifestations of COVID-19-associated coagulopathy have evolved as a result of immunity acquired through natural infection and vaccination and the emergence of new SARS-CoV-2 variants. Therefore, there is a need to reevaluate diagnostic criteria and management strategies for COVID-19-associated coagulopathy on the basis of updated experiences and studies (104). The corresponding thrombus prevention and treatment options are also changing. Therefore, we include the review of clinical trials and guideline recommendations.

### 3.1 Clinical trials

Through a systematic search of the Clinical Trials.gov and PubMed databases, we identified a total of 14 completed clinical trials on the use of anticoagulant drugs in COVID-19 patients (**Table 1**). Among them, only one clinical trial (NCT04354155) (105) has been conducted in children. The remaining clinical trials were conducted in adult patients. The COVID-19 study population was divided into nonhospitalized and hospitalized patients on the basis of the severity of the infection. Hospitalized patients were divided into noncritically ill and critically ill COVID-19 patients. Most of the clinical trials used the anticoagulant drugs unfractionated heparin (UFH)/low-molecular-weight heparin (LMWH). Interestingly, these trials focused on thrombosis prevention.

**Table 1:** Progress of clinical trials on prevention and treatment of thrombosis in patients with COVID-19.

Trial name (Trial ID)	Design	Population	Drug ang dose	Primary endpoint	Result	Conclusion	Reference
COVAC-TP (NCT04354155)	Phase 2 open-label single-arm	Children hospitalized for symptomatic COVID-19 (n=40)	Enoxaparin Initial dose: 0.5mg/kg bid; max: 60mg.	Efficacy: Cumulative incidence of VTE within 30 days post-discharge. Safety: ISTH-defined clinically-relevant bleeding (30 days).	No clinically relevant bleeding. Two children (5.3%) developed central-venous catheter-related venous thromboembolism. No serious adverse events.	Among children hospitalized for COVID-19, thromboprophylaxis with twice-daily enoxaparin appears safe.	(105)
ASPEN-COVID-19 (NCT04655586)	Multicenter RCT	People hospitalized with COVID‐19 (n=160)	Intervention: rNAPc2 (high/low dose) Control: Standard heparin	Efficacy: Proportional change in D-dimer (baseline to Day 8/discharge). Safety: Major or non-major clinically relevant bleeding (Day 8).	There was no significant difference in terms of safety between rNAPc2 and heparin.Median change in D-dimer was -16.8% (interquartile range, -45.7 to 36.8; P=0.41) with rNAPc2 treatment and -11.2% (-36.0 to 34.4; P=0.91) with heparin (Pintergroup=0.47).	rNAPc2 treatment in hospitalized patients with COVID-19 was well tolerated without excess bleeding or serious adverse events but did not significantly reduce D-dimer more than heparin at day 8.	(106)
HERO-19 (NCT04542408)	A prospective, multicenter, interventional Phase III trial	People with COVID‐19 (n=140)	Intervention: - Inpatient: Weight-adjusted therapeutic LMWH - Post-discharge: Edoxaban 60mg qd Control: - Inpatient: Prophylactic LMWH - Post-discharge: Placebo	Efficacy: 42-day all-cause death, VTE, or ATE.	Unknown	Unknown	–
FREEDOM COVID (NCT04512079)	3-arm open-label RCT	Noncritically ill patients hospitalized with COVID-19 (n=3398)	A: Apixaban 5mg bid B: Enoxaparin 1mg/kg bid C: Enoxaparin 40mg qd	Efficacy: 30-day composite: all-cause death, ICU need, thromboembolism, or ischemic stroke. Safety: Major bleeding (BARC types 3 or 5).	The 30-day primary outcome rate was 13.2% (in the preventive dose group) vs. 11.3% (in the therapeutic dose group) (HR: 0.85; 95% CI: 0.69 - 1.04; P = 0.11). The all-cause mortality rate was 7.0% vs. 4.9% (HR: 0.70; 95% CI: 0.52 - 0.93; P = 0.01), intubation rate was 8.4% vs. 6.4% (HR: 0.75; 95% CI: 0.58 - 0.98; P = 0.03). Intravascular hemorrhage (the primary safety endpoint) was not common, 0.1% VS 0.4%	Therapeutic anticoagulation did not reduce the 30-day composite outcome in noncritically ill COVID-19 inpatients vs. prophylactic dosing; however, it reduced intubation and mortality.	(107)
ACTION (NCT04394377)	Open-label RCT	Patients hospitalized with COVID-19 and elevated D-dimer concentration (n=3331)	Intervention: Rivaroxaban (20 mg or 15 mg qd) or enoxaparin (1 mg/kg bid)→rivaroxaban Control: Prophylactic heparin	Efficacy: Hierarchical composite: time to death, hospitalization duration, or oxygen use (30 days). Safety: Major or clinically relevant non-major bleeding.	Primary efficacy outcome: 34.8% (therapeutic) vs 41.3% (preventive) (win ratio 0.86 [95% CI 0.59 - 1.22], p = 0.40). Primary safety outcome: 5.8% (therapeutic) vs 2% (preventive) (relative risk 3.64 [95% CI 1.61 - 8.27], p = 0.0010).	In hospitalized COVID-19 patients with elevated D-dimer, therapeutic rivaroxaban or enoxaparin failed to improve 30-day outcomes and increased bleeding vs. prophylactic heparin. Avoid therapeutic oral anticoagulants without evidence-based indications.	(108)
ATTACC (NCT04372589)	A prospective, open-label, multicenter RCT	Noncritically Ill Patients with Covid-19 (n=1200)	enoxaparin 1.5 mg/kg qd or 1 mg/kg bid sc (14 days)	Efficacy: Mortality and days free of organ support	Unknow	Unknow	–
RAPID (NCT04362085)	A randomized controlled, adaptive, open label clinical trial	moderately ill patients with covid-19 admitted to hospital wards (n=465)	LMWH or UFH Intervention: Therapeutic dose of LMWH or high dose nomogram of UFH. Control: thromboprophylactic doses of LMWH or UFH.	Efficacy: 28-day composite of death, mechanical ventilation (invasive/non-invasive), or ICU admission. Safety: Major bleeding.	Main composite outcome: 16.2% (therapeutic heparin) vs. 21.9% (preventive heparin) (OR 0.69, 95% CI 0.43 - 1.10; P = 0.12). Major bleeding: 0.9% (therapeutic heparin) vs. 1.7% (preventive heparin) (OR 0.52, 95% CI 0.09 - 2.85; P = 0.69).	Therapeutic heparin did not reduce the 28-day composite outcome (death/mechanical ventilation/ICU admission) in moderately ill ward patients with elevated D-dimer but reduced 28-day mortality with low major bleeding risk.	(109)
ATTACC, ACTIV-4a, and REMAP-CAP (NCT04372589, NCT04505774, NCT04359277, NCT02735707)	An open-label, adaptive, multiplatform, randomized clinical trial	noncritically ill patients with Covid-19 (n=2219)	LMWH or UFH Intervention: Therapeutic-dose anticoagulation. Control: Thromboprophylaxis was provided at a dose and duration determined by the treating clinician according to local practice.	Efficacy: Organ support-free days Safety: Major bleeding.	the probability that therapeutic-dose anticoagulation increased organ support–free days was 98.6% (adjusted odds ratio, 1.27; 95% credible interval, 1.03 to 1.58). Major bleeding: 1.9% (treatment dose anticoagulation) vs 0.9% (usual-care thromboprophylaxis).	In noncritically ill patients with Covid-19, an initial strategy of therapeutic-dose anticoagulation with heparin increased the probability of survival to hospital discharge with reduced use of cardiovascular or respiratory organ support as compared with usual-care thromboprophylaxis.	(110)
ATTACC, ACTIV-4a, and REMAP-CAP (NCT04372589, NCT04505774, NCT04359277, NCT02735707)	An open-label, adaptive, multiplatform, randomized clinical trial	critically ill patients with severe Covid-19 (n=1098)	LMWH or UFH Intervention: Therapeutic-dose anticoagulation was up to 14 days or until recovery. Control: Usual-care thromboprophylaxis was administered at a dose and duration determined by the treating clinician according to local practice.	Efficacy: Organ support-free days Safety: Major bleeding.	(treatment dose anticoagulation) VS (usual-care thromboprophylaxis). The median value for organ support–free days:1 vs 4 (interquartile range, -1 to 16) (adjusted proportional odds ratio, 0.83; 95%CI, 0.67 to 1.03; posterior probability of futility [defined as an odds ratio <1.2], 99.9%). The percentage of patients who survived to hospital discharge was similar in the two groups (62.7% vs 64.5%, respectively; adjusted odds ratio, 0.84; 95% CI 0.64-1.11).	In critically ill patients with Covid-19, an initial strategy of therapeutic-dose anticoagulation with heparin did not result in a greater probability of survival to hospital discharge or a greater number of days free of cardiovascular or respiratory organ support than did usual-care pharmacologic thromboprophylaxis.	(111)
COVID-PACT (NCT04409834)	A multicenter, open-label, randomized-controlled trial	critically-ill hospitalized patients with COVID‐19 (n=390)	LMWH Intervention: Full-dose anticoagulation for prophylaxis Control: Standard-dose prophylactic anticoagulation	Efficacy: Composite VTE/ATE. primary safety outcome: fatal or life-threatening bleeding. secondary safety outcome: moderate to severe bleeding	12.3% (full-dose) vs 6.4% (standard-dose); win ratio, 1.95 [95% CI, 1.08 - 3.55]; P = 0.028). Primary efficacy endpoint: 9.9% (full-dose) vs 15.2% (standard-dose); HR, 0.56 [95% CI, 0.32 - 0.99]; P = 0.046). Primary safety endpoint: 2.1% vs 0.5% (P = 0.19); Secondary safety endpoint: 7.9% vs 0.5%; P = 0.002. There was no difference in all-cause mortality (risk ratio, 0.91 [95% CI, 0.56 - 1.48]; P = 0.70). There was no difference in the primary efficacy or safety endpoints between clopidogrel and no antiplatelet treatment.	Full-dose anticoagulation (without clopidogrel) reduced thrombotic complications but increased bleeding (primarily transfusions) without excess mortality in critically ill COVID-19 patients.	(112)
The HEP-COVID Randomized Clinical Trial (NCT04401293)	Open-label RCT	Critically-ill hospitalised patients with COVID-19 with D-dimer levels more than 4 times the upper limit of normal or sepsis-induced coagulopathy score of 4 or greater (n=257)	LMWH Intervention: Full dose LMWH anticoagulation therapy (enoxaparin). Control: Prophylactic/Intermediate dose LMWH or UFH therapy.	Efficacy: VTE, ATE, or all-cause death. Safety: Major bleeding (30 ± 2 days).	Primary efficacy endpoint: 41.9% (standard-dose) vs 28.7% (therapeutic-dose) (RR, 0.68; 95% CI, 0.49-0.96; P = 0.03) Reduction in thromboembolism: (29.0% vs 10.9%; RR, 0.37; 95% CI, 0.21-0.66; P < 0.001). The primary efficacy endpoint was reduced in non-ICU patients (36.1% vs 16.7%; RR, 0.46; 95% CI, 0.27-0.81; P = 0.004), but not in ICU patients (55.3% vs 51.1%; RR, 0.92; 95% CI, 0.62-1.39; P =0 .71). Major bleeding: 1.6% (standard dose) vs 4.7% (treatment dose) (RR, 2.88; 95% CI, 0.59 - 14.02; P = 0.17).	In this randomized clinical trial, therapeutic-dose LMWH reduced major thromboembolism and death compared with institutional standard heparin thromboprophylaxis among inpatients with COVID-19 with very elevated D-dimer levels. The treatment effect was not seen in ICU patients.	(113)
HESACOVID (REBEC RBR-949z6v)	A randomized, open-label, phase II study	Critically-ill hospitalised patients with COVID-19 requiring mechanical ventilation (n=20)	Enoxaparin The therapeutic enoxaparin group was allocated to receive subcutaneous enoxaparin. The standard thromboprophylaxis group was allocated to receive subcutaneous UFH at a dose of 5000 IU TID (if weight < 120 kg) and 7500 IU TID (if weight > 120 kg) or enoxaparin at a dose of 40 mg OD (if weight < 120 kg) and 40 mg BID (if weight > 120 Kg).	Efficacy: PaO₂/FiO₂ ratio change (baseline → Day 7/14). Safety: Major bleeding	The PaO2/FiO2 ratio over time in the therapeutic t enoxaparin group (163 [95% confidence interval – CI 133–193] at baseline, 209 [95% CI 171–247] after 7 days, and 261 [95% CI 230–293] after 14 days), p = 0.0004. prophylactic group (184 [95% CI 146–222] at baseline, 168 [95% CI 142–195] after 7 days, and 195 [95% CI 128–262] after 14 days), p = 0.487. The therapeutic group had a higher ratio of successful liberation from mechanical ventilation (hazard ratio: 4.0 [95% CI 1.035–15.053]), p = 0.031 and more ventilator-free days (15 days [interquartile range IQR 6–16] versus 0 days [IQR 0–11]), p = 0.028 when compared to the prophylactic group.	Therapeutic enoxaparin improves gas exchange and decreases the need for mechanical ventilation in severe COVID-19	(114)
NCT04360824	A prospective, open-label, multi-center RCT	People hospitalized with COVID‐19 and admitted to an intensive care unit (ICU) and/or had laboratory evidence of coagulopathy (n=176)	standard prophylactic dose enoxaparin (40 mg SC qd if BMI <30 kg/m2; 30 mg SC bid or 40 mg SC bid if BMI ≥ 30 kg/m2). intermediate-dose enoxaparin (1 mg/kg SC qd if BMI <30 kg/m2 or 0.5 mg/kg SC bid if BMI ≥ 30 kg/m2)	Primary outcome: all‐cause mortality at 30 days. Secondary outcome: arterial or venous thromboembolism and major bleeding	All‐cause mortality at 30 days:15% (intermediate dose enoxaparin)VS 21% (standard dose enoxaparin) (OR, 0.66;95%CI, 0.30-1.45;P =0.31 by Chi‐square test)。 Arterial or venous thrombosis:13% (intermediate dose enoxaparin)VS 9% (standard dose enoxaparin)。 Major bleeding occurred in 2% of patients in each arm.	In hospitalized adults with severe COVID‐19, standard prophylactic dose and intermediate dose enoxaparin did not differ significantly in preventing death or thrombosis at 30 days.	(115)
INSPIRATION (NCT04486508)	Open-label RCT	Patients with COVID-19 admitted to intensive care (n=562)	Intervention: Enoxaparin 1mg/kg qd Control: Enoxaparin 40mg qd	Efficacy: Composite: venous/arterial thrombosis, ECMO, or all-cause death. Safety: Major bleeding.	primary outcome:45.7% (intermediate-dose prophylactic anticoagulation)VS 44.1% (standard-dose prophylactic anticoagulation) (OR, 1.06).	Intermediate-dose compared with standard-dose prophylactic anticoagulation did not reduce a composite of death, treatment with ECMO, or venous or arterial thrombosis at 90-day follow-up. It not support routine empirical use of intermediate-dose prophylactic anticoagulation in unselected patients with COVID-19 admitted to the ICU.	(116)

ISTH, International Society Of Thrombosis And Haemostasis;rNAPc2, recombinant nematode anticoagulant protein c2; LMWH, low molecular weight heparin; SmPC, Summary of product characteristic;UFH, unfractionated heparin; CV, cardiovascular; VTE, Venous thromboembolism; ATE, arterial thromboembolism; SQ, subcutaneous injection; CrCl, creatinine clearance; CKD-EPI, Chronic Kidney Disease Epidemiology Collaboration; OD, once a day; ECMO, extracorporeal membrane oxygenation; SOC, standard of care.

In a single-arm clinical trial of enoxaparin for thrombosis prevention in hospitalized pediatric COVID-19 patients, the daily administration of enoxaparin for thrombosis prevention in children was found to be safe and worthy of further study to evaluate its efficacy (105).

In a clinical trial involving nonhospitalized COVID-19 patients, the incidence of cardiovascular events in the enoxaparin group was 0.9%, whereas that in the control group was 1.7% (relative risk 0.51; 95% confidence interval: 0.09–2.75). However, the early use of enoxaparin did not improve the course of the disease in patients with COVID-19 (117). In another nonhospitalized COVID-19 patient clinical trial, 8 patients who received enoxaparin experienced severe adverse reactions (118). A systematic review evaluated the efficacy of prolonged thromboprophylaxis in COVID-19 patients after discharge, including eight studies with a total of 10,148 patients. The results confirmed that prolonged thromboprophylaxis, mainly prophylactic use of anticoagulants for <35 days, was significantly associated with a poor overall prognosis in high-risk patients with COVID-19 after discharge (OR: 0.52; 95% CI: 0.41–0.67; P=0.000). Prolonged thromboprophylaxis did not increase the risk of major bleeding events (OR: 1.64; 95% CI: 0.95–2.82, P= 0.075) (119).

Most of the other clinical trials divided hospitalized patients into severe and nonsevere cases. One study compared two treatment regimens for noncritical hospitalized patients with COVID-19: one was a therapeutic dose of heparin anticoagulation therapy, and the other was a standard nursing drug for thrombosis prevention therapy. Among the 2219 patients ultimately included, initial therapeutic-dose heparin anticoagulant therapy improved the probability of survival to discharge while reducing the use of cardiovascular or respiratory support compared with conventional thromboprophylaxis. However, the incidence of major bleeding was greater in the group that received the therapeutic dose (1.9% vs. 0.9%) (110). Another trial compared patients with severe illness, with 1098 patients divided into 534 receiving treatment-dose anticoagulation and 564 receiving standard care thrombosis prophylaxis. The proportion of patients surviving to discharge was similar in both groups (62.7% and 64.5%, respectively; adjusted relative advantage, 0.84; 95% confidence interval, 0.64–1.11). A total of 3.8% of patients receiving treatment-dose anticoagulation had major bleeding, compared with 2.3% of patients receiving standard care thrombosis prophylaxis. In COVID-19 critically ill patients, the strategy of initial treatment-dose heparin anticoagulation compared with standard care pharmacological thrombosis prophylaxis did not result in a greater probability of survival to discharge or more days without cardiovascular or respiratory organ support (111). The NCT04360824 (115) and INSPIRATION (116) trials compared the efficacy and safety of different doses in critically ill patients and reported no significant difference in thrombotic or bleeding events between moderate and standard doses of enoxaparin. However, the therapeutic dose of enoxaparin in the HESACOVID trial (114) improved gas exchange and reduced the need for mechanical ventilation in patients with severe COVID-19. One meta-analysis included studies comparing therapeutic or intermediate anticoagulation versus prophylactic anticoagulation in COVID-19 patients, including 6 RCTs with 4678 patients and 4 cohort studies with 1080 patients. In randomized controlled trials, therapeutic anticoagulation or intermediate anticoagulation was associated with a significant reduction in thromboembolic events (5 studies, n=4,664; relative risk [RR], 0.72; P=0.01) and significantly increased bleeding events (5 studies, n=4,667; RR1.88; P= 0.004). In moderate patients, therapeutic or moderate anticoagulation was more beneficial than prophylactic anticoagulation for thromboembolic events, but the number of bleeding events was significantly greater. In severely ill patients, the incidence of thromboembolism and bleeding events occurs at or in the middle of treatment. It is suggested that patients with moderate and severe COVID-19 infection should be treated with preventive anticoagulation therapy (120). In another systematic review, which included 33 studies (11,387 patients), standard heparin prophylaxis versus high (intermediate or therapeutic) heparin regimens was evaluated in hospitalized patients with COVID-19. The incidence of VTE events was 5.2% and 8.2% with high- or standard-dose heparin prophylaxis, respectively (RR 0.71, 95% CI 0.55–0.90, 1248.8%). Major bleeding was significantly greater in patients receiving the high dose than in those receiving the standard dose (4.2% vs. 2.2%, RR 1.94, 95% CI 1.47–2.56, 1218.1%) (121).

Additionally, the COVID-PACT trial included critically ill patients and assessed the prevention of thrombosis with antiplatelet drugs. A total of 682 patients were enrolled, and this trial revealed that full-dose anticoagulation was superior to clopidogrel antiplatelet prevention for thrombosis, reducing the incidence of thrombotic events but increasing bleeding, which was mainly due to blood transfusions in patients with stable hemodynamics, with no significant increase in mortality. Owing to the decline in the incidence of ICU-level COVID-19, recruitment was discontinued in March 2022 (planned enrollment was 50%) (112). The HEP-COVID-19 multicenter randomized clinical trial assessed the efficacy of therapeutic doses of LMWH compared with standard prophylactic or medium-dose heparin in preventing COVID-19-related thrombosis in high-risk hospitalized patients. The trial also stratified patients on the basis of their ICU status, with 83 out of 257 randomized patients (32.8%) being in the ICU (113). Among the patients in the standard-dose group, 52 (41.9%) met the primary efficacy endpoint. In comparison, 37 (28.7%) patients in the therapeutic dose group met the primary efficacy endpoint. The incidence of thromboembolism decreased significantly from 29.0% to 10.9% (RR 0.37; 95% CI, 0.21–0.66; P < 0.001). Major bleeding occurred in 1.6% of the patients in the standard-dose heparin group and 4.7% of those in the therapeutic-dose heparin group (RR 2.88; 95% CI, 0.99–14.02; P = 0.17). The primary outcome measures were reduced in non-ICU patients (36.1% vs. 16.7%; RR 0.46; 95% CI, 0.27–0.81; P = 0.004), whereas no significant difference was observed in ICU patients (55.3% vs. 51.1%; RR 0.92; 95% CI, 0.62–1.39; P = 0.71). Compared with standard heparin thromboprophylaxis, therapeutic doses of low-molecular-weight heparin significantly reduced the incidence of major thromboembolism and mortality in hospitalized COVID-19 patients with markedly elevated D-dimer levels. Conversely, no therapeutic benefit was observed in patients admitted to the ICU.

Among the clinical trials described above, in addition to the heparin anticoagulant regimen, several trials evaluated the efficacy of new oral anticoagulants (NOACs) and a TF inhibitor in preventing blood clots in COVID-19 patients. rNAPc2 is a novel TF inhibitor with anticoagulant, anti-inflammatory, and potentially antiviral properties. Therefore, the ASPEN-COVID-19 trial utilized rNAPc2 to investigate whether TF could be a therapeutic target for COVID-19 thrombus prophylaxis. Among patients hospitalized with COVID-19, TF inhibition with rNAPc2 as the only anticoagulant was safe and well tolerated but did not significantly reduce D-dimer levels more than the heparin standard of care. The NCT04542408 trial evaluated edoxaban, but the trial data and results were not published. In the FREEDOM COVID anticoagulation strategy randomized trial, 3398 noncritically ill patients hospitalized for COVID-19 were randomly assigned to receive a prophylactic dose of enoxaparin (n=1141), a therapeutic dose of enoxaparin (n=1136), or a therapeutic dose of apixaban (n=1121). The results were similar in both treatment dose groups, with major bleeding uncommon in all three groups (107). ACTION is an open-label, multicenter, randomized, controlled trial: patients (aged ≥18 years) who were hospitalized with COVID-19 and had elevated D-dimer concentrations and symptoms of COVID-19 for up to 14 days prior to randomization were randomly assigned (1:1) to receive therapeutic or preventive anticoagulant therapy. Therapeutic anticoagulant therapy was oral rivaroxaban at hospitalization (20 mg or 15 mg daily) for stable patients and an initial subcutaneous injection of enoxaparin (1 mg/kg twice daily) or intravenous injection of UFH (target 0-3-0–7 IU/mL anti-Xa concentration), followed by rivaroxaban until day 30, for clinically unstable patients. There was no difference in primary efficacy outcomes among patients receiving therapeutic and preventive anticoagulant therapy. Compared with prophylactic anticoagulation, in-hospital anticoagulation treatment of up to 30 days of rivaroxaban or enoxaparin did not improve clinical outcomes, and bleeding increased (108).

It is perplexing that various anticoagulants exhibit differing therapeutic effects across patient subgroups stratified by disease severity. The author of this article hypothesizes that there are several possible reasons. The pathophysiological mechanism of the patients undergoes dynamic changes. For mild/moderate patients, it is mainly characterized by vascular endothelial micro-inflammation and local hypercoagulability, with limited thrombin production. Therefore, the effect of heparin-like drugs is limited. While for severe patients, “immune thrombosis” occurs (immunothrombosis), NETs are released and systemic coagulation activation takes place. In addition to their anticoagulant effect, using heparin can also neutralize the histones of NETs (through negative charge binding), exerting an anti-inflammatory effect. However, DOACs do not have this effect. From the perspective of pharmacokinetics, there are differences among patients with varying degrees of severity.

### 3.2 Related guidelines

To address the risk of thrombosis in patients with COVID-19, many academic groups in China and abroad have issued relevant guidelines for the prevention and treatment of thrombosis in COVID-19 patients. Most guidelines refer to anticoagulant therapy with heparin or low-molecular-weight heparin, but only a few guidelines mention direct oral anticoagulant (rivaroxaban) (122, 123).

Nonhospitalized COVID-19 patients: The ISTH guidelines define those who are “not hospitalized” as people with COVID-19 who live in the community and have not recently been hospitalized for COVID-19 (123). Guidelines suggest that initiating direct oral anticoagulant therapy or antiplatelet therapy does not effectively reduce the risk of hospitalization, arterial or venous thromboembolism, or death in symptomatic COVID-19 patients who are not hospitalized. In nonhospitalized COVID-19 patients at increased risk of disease progression, the initiation of oral sulodexide treatment within 3 days of symptom onset may be considered to reduce the risk of hospitalization. In asymptomatic COVID-19 patients, LMWH for preventing blood clots is not effective in reducing the risk of disease progression (123).

Noncritically ill patients hospitalized for COVID-19: For inpatients, most foreign guidelines classify hospitalized nonpregnant adult patients with COVID-19 without evidence of VTE into two categories: noncritically or acutely ill patients hospitalized for COVID-19 (in the general inpatient ward) and critically ill patients hospitalized for COVID-19 (in the ICU). Low-dose (prophylactic/standard) LMWH or UFH should be used in noncritically ill patients hospitalized for COVID-19 to reduce thromboembolism and the possible risk of death, and it is recommend that clinicians consider the therapeutic intensity of LMWH or UFH thromboprophylaxis in noncritically ill patients at increased risk of disease progression or thromboembolism and not at high risk of anticoagulation-related bleeding (123). In the Guidelines for diagnosis, prevention and treatment of COVID-19 in adults in China, anticoagulation prevention is also recommended for mild and general COVID-19 patients with medical or surgical conditions who are assessed as having a high or medium-high risk of VTE on the basis of the Padua score or Caprini score and who have no anticoagulation conjunctivitis (124). The ASH guidelines also recommend the use of anticoagulant therapy with standard prophylactic intensity over therapeutic intensity for patients with COVID-19-related acute illness without suspected or confirmed VTE or other anticoagulant indications (125). The NIH guidelines further recommend the use of therapeutic doses of heparin in patients with D-dimer levels above the upper limit of normal who require low oxygen flow and are not at increased risk of bleeding (126).

Critically ill hospitalized patients with COVID-19: In the absence of contraindications, all guidelines recommend anticoagulant prophylaxis in critically ill hospitalized patients with COVID-19. All guidelines (including domestic guidelines) recommend the use of a prophylactic dose for patients with COVID-19-related critical illness who do not have suspected or confirmed venous thromboembolism. For critically ill patients with COVID-19, the CHEST guidelines (127, 128) recommend against routine ultrasound screening for asymptomatic DVT and adding mechanical prophylaxis to drug thrombus prophylaxis. CHEST guidelines recommend mechanical thromboprophylaxis if drug thromboprophylaxis is contraindicated in critically ill COVID-19 patients. In addition, the domestic guidelines make other recommendations (129). For hospitalized patients with severe COVID-19, if there is a high risk of VTE and a low risk of bleeding and the disease has a tendency to worsen (such as a progressive increase in D-dimers and exacerbation of hypoxemia), the use of a therapeutic dose of LMWH/UFH anticoagulation is recommended. If bleeding is a high risk, mechanical prophylaxis is recommended. After the risk of bleeding is reduced, drug prophylaxis combined with mechanical prophylaxis should be carried out in time. The Anticoagulation Forum Guidelines (122) suggest that posthospital thromboprophylaxis with 10 mg of rivaroxaban daily for 35 days following hospitalization for COVID-19 may be considered in select patients at increased risk of thromboembolism (e.g., IMPROVE VTE score ≥ 4 or score 2–3 with elevated D-dimers at hospital discharge) and not at increased risk of bleeding, regardless of the intensity of their inpatient thromboprophylaxis.

Discharged patients: All guidelines recommend against routinely continuing VTE prophylaxis in patients with COVID-19 after hospital discharge unless they have another indication for anticoagulation. According to the Guidelines for Thrombosis Prevention and Anticoagulation Management in Hospitalized Patients with Novel Coronavirus Infection (129), if a high risk of VTE and low risk of bleeding are assessed before discharge, drug prophylaxis with NOACs can be recommended. If a high risk of VTE and a high risk of bleeding are assessed before discharge, mechanical prophylaxis (such as GCS) or early activity is recommended. For hospitalized patients with COVID-19, if out-of-hospital prophylaxis is assessed before discharge, prophylaxis should be prolonged depending on the risk of VTE or until the risk factors are removed. The ISTH guidelines (123) suggest that in select high-risk patients who have been hospitalized for COVID-19, postdischarge treatment with prophylactic-dose rivaroxaban for approximately 30 days may be considered to reduce the risk of major thromboembolism. In patients who have been hospitalized for COVID-19 and are not deemed at high risk for complications, routine postdischarge prophylactic doses of direct oral anticoagulants are not recommended to reduce the risk of death or thromboembolism.

For antithrombotic treatment, the CHEST guidelines (127, 128), Anticoagulation Forum guidelines (122) and ‘Guidelines for Thrombosis Prevention and Anticoagulation Management in Hospitalized Patients with Novel Coronavirus Infection’ refer to anticoagulation therapy for COVID-19 patients with thromboembolism. It is recommend that most patients who are diagnosed with VTE while hospitalized for COVID-19 receive anticoagulation therapy for a minimum of three to six months. For critically ill COVID-19 patients with proximal DVT or PE, we suggest parenteral rather than oral anticoagulant therapy. For critically ill COVID-19 patients with proximal DVT or PE who are treated with parenteral anticoagulation, we suggest LMWH or fondaparinux over UFH. In patients with COVID-19 and recurrent VTE despite anticoagulation with apixaban, dabigatran, rivaroxaban or edoxaban (and documented compliance) or vitamin K antagonist therapy (in the therapeutic range), we suggest switching treatment to therapeutic weight-adjusted LMWH.

## 4 Long COVID-19/postacute sequelae of COVID-19

COVID-19 can lead to a variety of pathophysiological changes and clinical symptoms. Although symptoms usually last only 2–3 weeks, after the acute phase, approximately 10% of patients develop persistent or new symptoms, a condition known as long COVID-19/postacute sequelae of COVID-19 (130). Long COVID-19 has the potential to impact multiple organ systems, resulting in significant and lasting functional impairments attributable to organ damage. The resulting burden of this disease on individuals, healthcare systems, and national economies is substantial (131). Cardiovascular complications (including thromboembolic diseases) have emerged as a key issue in PASC (132, 133). So far, the main potential pathophysiological mechanisms related to vascular complications include immune dysregulation, autoimmunity, endothelial dysfunction (ED), coagulopathy, etc. Although the acute phase of cardiovascular complications is characterized by excessive inflammation and coagulation dysfunction, the mechanisms driving the persistent cardiovascular dysfunction in PASC remain poorly understood (134). Furthermore, numerous procoagulant inflammatory molecules have been identified within microclots associated with long COVID-19. These molecules include α2-antifibrinolytic protein (α2AP), von Willebrand factor (vWF), platelet factor 4 (PF4), serum amyloid A (SAA), various fibrinogen chains, and a range of antibodies (1). Notably, circulating NET biomarkers do not return to normal levels until approximately 4 months after infection (135). In cases of long COVID-19, feedback loops involving NETs and thrombosis establish a cycle characterized by coagulation, inflammation, and localized hypoxia, which contributes to the severity of COVID-19 and the persistence of long-term symptoms.

New evidence suggests that mitochondrial dysfunction within immune cells may play a crucial role in the pathogenesis of PASC, especially in relation to cardiovascular sequelae (136). Mitochondria play a crucial role in the generation of cellular energy, the function of immune cells, regulation of oxidative phosphorylation (OXPHOS), production of reactive oxygen species (ROS), and cell apoptosis, among other processes. (137) Mitochondria are usually the targets of viral proteins, which can lead to disruptions in biological energy and changes in the immune response (138). Meanwhile, monocytes also rely heavily on mitochondrial function to maintain innate immunity and vascular homeostasis. Mitochondrial damage can also lead to a decline in monocyte function (139). In long-term COVID patients, the persistent oxidative stress leads to continuous mitochondrial damage and cellular dysfunction, thereby perpetuating the cycle of inflammation and tissue damage (140, 141). Furthermore, genetic factors and genetic polymorphisms may also contribute to an increased susceptibility of long COVID patients to vascular complications (142). For example, the polymorphisms of genes related to blood coagulation, F5 (R506Q) and F2 (G20210A), can increase the susceptibility to thromboembolic events by enhancing procoagulant activity or reducing anticoagulant regulation. In COVID-19, these polymorphisms work in synergy with a high inflammatory state, resulting in excessive thrombus formation in the pulmonary vessels, which leads to pulmonary thromboembolism (143, 144).

In patients with long-term COVID-19, the routine use of anticoagulants or antiplatelet drugs for thrombosis prevention is not recommended (145). A Korean guideline recommends treatment with anticoagulants or antiplatelet agents according to the relevant guidelines for patients with long COVID-19 diagnosed with thrombosis (146).

## 5 COVID-19 vaccine

Overall, the currently approved COVID-19 vaccines are effective in reducing the incidence and mortality rates associated with the disease (147). Real-world studies show that vaccination reduces the risk of COVID-19 hospitalization by 85-95% and the risk of severe disease by 91% (Delta variant era data) (148). Although vaccines may cause extremely rare blood clots (such as VITT, with an incidence of approximately 1 in 250,000 doses) (149), the risk of blood clot caused by COVID-19 itself is even higher (about 16.5% of hospitalized patients develop VTE) (150). Mathematical models show that vaccination can prevent more than 40 times the number of thrombosis-related deaths (151). A study analyzed the VTE cases of 792,010 patients who received the authorized COVID-19 vaccine in the United States. The results showed that there was no increase in the risk of VTE after vaccination compared to before vaccination (152). That is to say, the benefits of global vaccination for individuals and the public far outweigh the adverse effects of the vaccines (153).

Nevertheless, the potential risk of thromboembolic events associated with vaccination continues to warrant careful consideration. Studies have shown that patients who received SARS-CoV-2 vaccines (such as ChAdOx1 or nCoV-19) developed thrombosis or thrombocytopenia, which is referred to as vaccine-induced immune thrombotic thrombocytopenia (VITT) (154). As a result, many countries have restricted their use in young patients. The main mechanisms include that Anti-PF4 antibody-mediated platelet activation, vaccine components trigger immune responses, and adenovirus vector vaccines may release free DNA or negatively charged proteins (such as spike proteins), which combine with platelet factor 4 (PF4) to form PF4-polyanion complexes (155). Anti-PF4 antibodies can also activate platelets through the FcγRIIa receptor, leading to platelet aggregation, thrombosis, and secondary thrombocytopenia (156). In addition, autoantibodies are produced, and some individuals develop anti-PF4 antibodies (similar to heparin-induced thrombocytopenia, HIT), but unlike HIT, VITT patients usually have no history of heparin exposure (157). Activated platelets release procoagulant microparticles, and endothelial injury triggers the expression of TF, promoting widespread thrombosis (158).

The primary intervention for VITT management is immunomodulatory therapy with intravenous immunoglobulin (IVIG) (155). It can rapidly increase platelet count (usually within 24–48 hours) and reduce the risk of thrombosis progression (159). If IVIG is ineffective or in severe cases (such as multiple organ thrombosis), glucocorticoids can be used as an adjunctive treatment (160). Anticoagulation therapy should prefer non-heparin anticoagulants(typically argatroban infusion or fondaparinux) (161). Additionally, platelet transfusion should be avoided unless there is life-threatening bleeding, and it should be used after IVIG (17). For refractory VITT, intensified treatment can also consider plasma exchange (TPE) (161).

## 6 Discussion

In general, we have an increasing understanding of the mechanism of COVID-19-induced thrombosis from existing studies, which can provide more references for medical workers. The guidelines also have updated recommendations based on clinical studies. The authors of this review suggest that the prevention of thrombosis in COVID-19 patients requires the timely assessment of their thrombosis and bleeding risk, clarification of any anticoagulation contraindications, and the use of diagnostic tests to evaluate their hypercoagulable or fibrinolytic state to better apply treatment strategies. We also found several interesting points when conducting this review.

Besides COVID-19, infections resulting from other respiratory diseases may also increase the risk of thrombotic events. a case report of a 58-year-old man with confirmed influenza virus infection who presented with deep vein thrombosis and acute pulmonary embolism (162). A review of 58 patients with laboratory-confirmed influenza A or B and associated intravascular thrombosis revealed 21 cases of pulmonary embolism (36.2%), 12 cases of DVT (20.6%) and 3 cases of DVT with pulmonary embolism (5.1%) (163). Similar thrombotic complications were observed in patients with severe acute respiratory syndrome (SARS) (164). In a nationwide study conducted from 2012–2014, the incidence of thrombosis among patients hospitalized with acute viral respiratory illnesses, excluding COVID-19, was compared to the incidence among patients hospitalized with COVID-19 within a large health system in New York. That study revealed that 1.6% of patients with non-COVID-19 viral respiratory illnesses also experienced venous thromboembolism. Furthermore, from 2002 –2014, the proportion of hospitalized patients with viral respiratory diseases who developed thrombosis was significantly lower than that of patients with COVID-19 (5% versus 16%; P < 0.001) (165). A further literature search revealed many similarities and differences between influenza and COVID-19. Comorbidities such as cardiovascular disease, diabetes, and obesity were significantly more common in COVID-19 patients, whereas lung disease and immunocompromised status were significantly more common in influenza patients. In terms of mechanism, both influenza and COVID-19 can cause a cytokine storm, which further stimulates inflammatory immunity to promote thrombogenesis (166). That study revealed that TF expression was upregulated in lung epithelial cells, with concomitant increases in EVTF activity and coagulation activity in bronchoalveolar lavage fluid (BALF) following influenza A virus infection in murine models. Similarly, mice infected with MERS-CoV, SARS-CoV, or SARS-CoV-2 presented elevated lung TF expression. Furthermore, patients suffering from severe respiratory tract infections demonstrated high levels of TF expression, strongly indicating that this upregulation may contribute to the pathogenesis of thrombosis (167).

The multinational and multicenter data included in this review summarize the clinical trials of anticoagulant therapy for COVID-19 patients. The differences in the studies may result from the different definitions, diagnostic methods and preventive management methods of all the included studies. In clinical trials, low-molecular-weight heparin/common liver and kidney drugs are often used, and there are a few mentions of new oral anticoagulants. The reason might be that anticoagulants such as heparin, LMWH or UFH have anti-inflammatory properties, which can inhibit the formation of thrombin and reduce inflammatory responses (168). The expected effect of heparin may also disrupt the lung-protective process of blood clotting, which could increase the survival rate of the host in COVID-19 (168). Furthermore, the antiviral efficacy of heparin explains why it prevents entry of the SARS-CoV-2 virus by acting on the ACE-2 receptor and interacting with the SARS-CoV-2 spike glycoprotein (169).

In addition to clots caused by the previously described immunoinflammatory mechanisms, D-dimers are also of concern, with elevated D-dimers being the most common feature of clotting diseases associated with COVID-19. The value of D-dimers in predicting the risk of VTE in COVID-19 patients has been demonstrated by numerous clinical studies, such as a multicenter study in France that revealed that the cooccurrence of elevated D-dimers (>3000 μg/L) and elevated white blood cell count (≥12.0×10^9^/L) was significantly associated with PE. Among many clinical laboratory indicators, D-dimers have the highest predictive value for VTE. In addition to the acute phase of infection, studies have shown that COVID-19 patients with higher peak D-dimer levels (>3000 μg/L) have an approximately fourfold increased risk of VTE after being cured and discharged (170). In clinical trials, some of which also included patients with moderate COVID-19, therapeutic heparin was not significantly associated with a reduction in the primary outcome in moderate patients hospitalized with COVID-19 and elevated D-dimer levels but was associated with a reduced chance of death within 28 days; in the trial, the risk of major bleeding appeared to be low (109). In addition to existing anticoagulant or antiplatelet agents, nematode anticoagulant protein c2 (rNAPc2), a potent TF inhibitor, was investigated in the ASPEN-COVID-19 trial. rNAPc2 was found to be well tolerated in hospitalized COVID-19 patients with no excessive bleeding or serious adverse events, but its ability to reduce the level of D-dimers was not significantly greater than that of heparin at day 8 (106). Fibrin amyloid microclots have been extensively observed in cases of long-term SARS-CoV-2 infection, which causes the disease known as long COVID-19. These microclots have the capacity to obstruct capillaries, consequently impeding the passage of red blood cells and hindering oxygen exchange. This obstruction can result in the formation of microthrombi. Consequently, the development and careful monitoring of anticoagulation and antiplatelet treatment regimens in randomized clinical trials could present a viable therapeutic strategy (171). In a similar manner, fibrin interacts with the SARS-CoV-2 spike proteins to form proinflammatory clots, which contribute to systemic thromboinflammation and neuropathology in COVID-19 patients. A monoclonal antibody that targets the inflammatory domain of fibrin has been shown to protect against microglial activation, neuronal injury, and thrombotic inflammation in the postinfection lung. Consequently, fibrin plays a pivotal role in the inflammation and neuropathology associated with SARS-CoV-2 infection, suggesting that fibrin-targeted immunotherapy could serve as a potential therapeutic intervention for patients experiencing both acute and long-term effects of COVID-19 (172). These findings also provide some ideas for our future research direction. We look forward to the results of more studies in the future and advancements in the guidelines.
